# Supplementary material for: Surviving to Acute Myocardial Infarction: The Role of Psychological Factors and Alexithymia in Delayed Time to Searching Care: A Systematic Review
Source: J Clin Med. 2021 Aug 25;10(17):3813. doi: 10.3390/jcm10173813 (PMC8432253; doi:10.3390/jcm10173813)
Supplement: Supplementary file 1 [file jcm-10-03813-s001.zip › jcm-1315221-supplementary.pdf]

Table S1. Included studies

FIRST AUTHOR AND YEAR OF PUBLICATION [reference]	COUNTRY	STUDY DESIGN	SAMPLE SIZE	PREHOSPITAL DELAY (PHD) or PATIENT DECISIONAL DELAY (PDD	CUT-OFF DELAY TIME (case-control studies)	PSYCHOLOGICAL FACTORS ASSOCIATED WITH PHD or PDD	MACRO-CATEGORIES
Abed et al., 2015 [37]	Jordan	Cross-sectional	299	PHD: mean±sd=7.5±18.4 hours OK		Symptoms incongruence (mismatch between AMI experienced symptoms and expected ones); perceived AMI symptoms as not serious; anxiety	Symptoms appraisal
Albarqouni et al., 2016a [57]	Germany	Survey	592	PHD: median=200 (range 100–684) minutes OK		Fear of death	Other (fear of death)
Al-Hassan & Omran., 2005 [36]	Jordan	Cross-sectional	79	PDD: mean±sd =354±1265, minutes OK	> 2 hours (delayers)	Perceived seriousness about symptoms	Symptoms appraisal
Bleeker et al., 1995 [51]	Netherlands	Case-control	300	PDD: media = ~ 30 minutes OK	>30 minutes (delayers)	Ignoring the symptoms; seeking distraction instead of social support; misinterpretation of symptoms as of cardiac origin; emotional denial	Coping strategies
Burnett et al., 1995 [38]	US	Case-control	453	PDD: mean±sd = 3.05±4.97 hours OK	≥ 60 minutes (delayers)	Attribution of symptoms to a non-cardiac origin; symptoms perceived as relatively mild; feeling less anxious or upset when symptoms were noticed and much control of the symptoms	Symptoms appraisal
Cartabellotta et al., 2020 [19]	France	Case-control	481 (among them, 271 answering a questionnaire also on psychological factors to be supposed impacting PDD)	Among 481 STEMI patients, PDD: median=87 (range: 0-1397) minutes OK	≥ 154 minutes (delayers: median: 309 (range: 215.25-493.25) minutes; early callers: median 50 (range: 24.5-86.5) minutes).	Among 481 with available data from the clinical reports: the initial care provider different from Emergency Medical Service at the first symptoms presentation.<br>Among 271 answering the questionnaire: the absence of sweats; the patient feeling that the situation was not severe.	Symptoms appraisal/Coping strategies
Carney et al., 2002 [35]	Ireland	Survey	62	PHD: mean±sd=2388.71±1083.55 minutes OK		Attribution of symptoms to a non-cardiac origin; cardiac denial; chance health locus of control	Symptoms appraisal
Carta et al., 2013 [56]	Italy	Case-control	83	PHD: mean±sd=176.75±239.65 minutes OK	> 120 minutes (delayers)	Alexithymia; contact with primary care before hospitalization	Alexithymia

Clark et al., 1992 [24]	US	Case-control	315	PHD: mean±sd=11.9±25.1 hours OK	> 4 hours (delayers)	Wrong beliefs about symptoms etiology (i.e.: "Thought symptoms might not be a heart attack)	Symptoms appraisal
Dracup et al., 1997 [26]	Australia	Survey	317	PHD: median=6.4 (interquartile range, 1.9-19.9) hours OK		Waiting to see if symptoms would go away; being too embarrassed to ask for an ambulance; being worried about troubling others; fear of what might happen if assistance was sought; knowing the nature of cardiac symptoms; failing to recognize the gravity of symptoms	Symptoms appraisal/coping strategies
Dracup et al., 2003 [52]	Australia, Japan, Korea, UK, US	Cross-sectional	913	PHD: mean±sd=17.3±45 hours OK		Wishing or praying for symptoms to go away; trying to relax; pretending nothing is wrong; trying not to think about the problem; taking medication; calling the physician; trying self-help remedy; telling someone nearby; calling EMS; going to **a** hospital without emergency medical services	Coping strategies
Dracup & Moser, 1997 [25]	US	Survey	277	PHD: mean±sd=110±79 minutes OK		Cognitive and Emotional responses (symptoms assessed as intermittent; appraisal of the symptom as not serious; Attribution of symptoms to a non-cardiac origin; symptoms' underestimation; waiting for symptoms to go away; worrying about troubling others and refraining from seeking help; fear of the consequences of seeking help)	Symptoms appraisal/coping strategies
Fang et al., 2016 [54]	Germany	Cross-sectional	533	PHD: median=203 (range: 101.5-695.0) minutes OK		Denial	Coping strategies
Fox-Wasylyshyn et al., 2010 [48]	Canada, US	Cross-sectional	135	PDD: median=2 mean±sd=11.8±23.7 (hours) OK		Attribution of symptoms to a non-cardiac origin and emotion-focused coping	Symptoms appraisal/Coping strategies
Fukuoka et al., 2005 [27]	Japan	Cross-sectional	145	PHD: median=3.34 hours OK		Interdependent construal of the Self	Symptoms appraisal
Horne et al., 2000 [29]	UK	Cross-sectional	88	PHD: mean±sd=7.3±14.2 (hours) OK		Mismatch between expected symptoms and experienced symptoms	Symptoms appraisal
Hwang & Jeong, 2012 [41]	Korea	Case-control	165	PHD: median=12 hours OK	> 6 hours (delayers)	Attribution of symptoms to a non-cardiac origin; presence of preinfarction angina pain	Symptom appraisal
Kentsch et al., 2002 [49]	Germany	Case-control	739	PHD: median=180 minutes (mean=622 minutes, range=4 minutes-7 days) OK	> 1 hours (delayers)	Emotional attitudes to AMI symptoms and inadequate coping strategies: waiting and see; symptoms gravity underestimation; worries about troubling others; Apparent improvement of the symptoms; asking others for advice; taking pain medication	Coping strategies
Kenyon et al., 1991 [55]	US	Cross-sectional	103	PHD: mean±sd=9±10.8 hours OK		Somatic nonawareness; emotional nonawareness (alexithymia)	Alexithymia
Khan et al., 2007 [40]	Pakistan	Cross-sectional	720	PHD: mean±sd=12.3±1.7 hours OK		Lack of knowledge about symptoms of heart attack, low perceived severity of chest pain.	Symptom appraisal

Khraim et al., 2009 [32]	Jordan	Survey	134	PDD: mean±sd=6.6±14.3 hours OK		Waiting to see if symptoms would go away; perceived symptom seriousness; anxiety due to continued symptom presentation; embarrassment about getting help; worries about the possible outcome; worries about troubling others.	Symptoms appraisal
Lesneski, 2010 [46]	US	Cross-sectional	105	PHD: mean±s=5.7±1.6 hours OK		Misinterpretation of symptoms as **of** cardiac origin, having a low pain scale, thinking to be in control of the symptoms (i.e.: taking a medication, resting, trying to relax, praying, or wishing symptoms would go away)	Symptoms appraisal/coping strategies
Li & Yu, 2017 [44]	Hong Kong	Cross-sectional	301	PHD: median=3.0 (interquartile range: 1.84-10.30) hours OK		Failed attribution of symptoms to AMI; not being aware of one's own susceptibility to the condition; not having a good understanding of the manifestation of AMI	Symptoms appraisal
Li & Yu, 2018 [45]	Hong Kong	Cross-sectional	301	PHD: median=3.0 (interquartile range: 1.84-10.30) hours OK		Perceived barriers to care seeking; poor congruence about AMI symptoms expectations; few typica AMI symptoms.	Symptoms appraisal/coping strategies
McKinley et al., 2000 [30]	US, Canada, Australia	Cross-sectional	424	PHD: median=90 minutes OK	> 6 hours (delayers)	Underestimation of the gravity of symptoms; waiting for symptoms to go away; worries about troubling others; attribution of symptoms to a non-cardiac origin; Attribution of symptoms to a non-cardiac origin; fearing the consequences of seeking help; embarrassment about seeking help	Symptoms appraisal
McKinley et al., 2004 [31]	US, South Korea, Japan, England	Survey	595	PHD: median (interquartile range): US 3.5 (1.2–15.2) hours; South Korea 4.4 (1.8–13.3) hours; Japan 4.5 (2.0–16.3) hours and England 2.5 (1.5–8.7) hours OK		Attribution of symptoms to a cardiac origin and not waiting for symptoms to go away	Symptoms appraisal
Meloni et al., 2016 [47]	Italy	Cross-sectional	95	PDD: mean=159 minutes, range 84-450 (alexithymic subjects N 27); mean=35 minutes, range 19–63 (non-alexithymic subjects N 68) OK		High alexithymia; waiting for symptoms to go away.	Alexithymia/Coping strategies
Momeni et al., 2012 [43]	Iran	Cross-sectional	162	PHD: mean±sd=7.4±16.25 hours OK		Misinterpretation of symptoms as of cardiac origin; perception of symptoms as not serious.	Symptoms appraisal
Morgan, 2005 [42]	US	Cross-sectional	98	PDD: median=93 minutes OK		Mismatch between experienced symptoms and expected ones	Symptoms appraisal
Nymark et al., 2019 [39]	Sweden	Cross-sectional (with a Case-control part)	306	PDD: median=3 (interquartile range 0.7–24) hours OK	< 1 hour (short delayers); > 12 hours (long delayers)	Perceived inability to act (i.e.: became paralyzed, lost control of themselves, became powerless, being frustrated and or unable to act despite persisting symptoms) and perceived symptom as not enough severe to seek medical care.	Symptoms appraisal/coping strategies

O'Carrol et al., 2001 [34]	UK	Case-control	72	PHD: mean = 474.7; median=167 (minutes) OK	> 4 hours (delayers)	Cardiac denial; "external" locus of control; neuroticism	Symptoms appraisal/Alexithymia
Perry et al., 2001 [33]	Not reported	Cross sectional	47	PHD: mean±se =15.3±4.1; median=4 (hours) OK		Mismatch between experienced and expected symptoms; talking with someone during symptom onset.	Symptoms appraisal
Song et al., 2010 [23]	China	Survey	799	PH: median=140 (interquartile range=75-300) minutes OK		Symptoms interpretation as non-cardiac in origin	Symptoms appraisal
Walsh et al., 2004 [53]	Ireland	Cross-sectional	61	PHD: median=244 (interquartile range 86–325) minutes OK		Low perceived threat; lack of emotional response (i.e.: anxiety, tense, startled); lack of active-cognitive coping style; lack of problem-focused coping style	Coping strategies
Zegrean et al., 2009 [50]	Canada, US	Cross-sectional	135	PDD: mean±sd=2±23.7 hours OK		Coping strategies: trying to relax; wishing/praying for symptoms to disappear; discussing symptoms with someone; trying not to think about symptoms; taking nonprescription medications; trying other home remedies; doing something to take one's mind off the symptoms; engaging in normal activities; persuading oneself and others that the problem is not serious	Coping strategies
Zhang et al, 2020 [58]	China	Cross-sectional	256	PHD: median=150 minutes OK		Type D Personality tends to increase awareness of the severity of AMI symptoms in female (not in male) patients, leading to less time spent on making decisions to seek care.	Other (Type D personality)
